# Multi-site microbiomes’ response to chronic obstructive pulmonary disease

**DOI:** 10.1128/spectrum.00007-26

**Published:** 2026-05-18

**Authors:** Mingxuan Liu, Wei Zhang, Jing Zhang, Na Lv, Xiukun Wu, Adel I. Alalawy, Wenfan Zhao, Hairong Bao, Jianjun Wu

**Affiliations:** 1 School of Public Health, Gansu University of Chinese Medicine 381940 , Lanzhou, China; 2 The First School of Clinical Medicine, Lanzhou University 12426, Lanzhou, China; 3 School/Hospital of Stomatology, Lanzhou University 12426, Lanzhou, China; 4 The Second Hospital & Clinical Medicine School, Lanzhou University 12426, Lanzhou, China; 5 Key Laboratory of Extreme Environmental Microbial Resources and Engineering 34737 , Lanzhou, China; 6 Engineering/State Key Laboratory of Ecological Safety and Sustainable Development in Arid Lands, Northwest Institute of Eco-Environment and Resources, Chinese Academy of Sciences 34737 , Lanzhou, China; 7 Department of Biochemistry, Faculty of Science, University of Tabuk 125900, Tabuk, Kingdom of Saudi Arabia; Nova Southeastern University 2814, Fort Lauderdale, Florida, USA

**Keywords:** chronic obstructive pulmonary disease, microbial dysbiosis, oral-lung-gut axis, full-length 16S rRNA gene sequencing

## Abstract

This study aimed to evaluate changes in the oral, nasal, pulmonary, and gut microbiota in patients with chronic obstructive pulmonary disease (COPD) and to explore their interrelationships compared with the healthy group. This study included 33 COPD patients and 29 healthy individuals. A total of 162 oral, nasal, sputum, and fecal samples were obtained. The microbiota was determined using full-length 16S rRNA gene sequencing on the PacBio platform. Alpha diversity was significantly reduced in sputum and fecal samples of COPD patients, while oral and nasal microbiota showed no significant differences. Beta diversity revealed substantial overlap between oral and sputum microbiota in both groups, while nasal and fecal communities were clearly distinct. Linear discriminant analysis effect size analysis identified *Haemophilus parahaemolyticus* as a sputum biomarker. Source tracking confirmed that the majority of lung microbiota originated from the oral cavity. Interleukin-6 was inversely correlated with short-chain fatty acids (SCFAs)-producing microbiota in fecal samples, suggesting that depletion of these bacteria may contribute to systemic inflammation. Co-occurrence network analysis revealed that the sputum microbial network in COPD patients exhibited reduced robustness and lacked prominent hub nodes. Lung microbiota largely originates from the oral cavity but is changed in COPD. The lung microbiome is still more sensitive and accurate than the oral, nasal, and fecal microbiomes for COPD diagnosis. Fragmented networks in COPD indicate reduced community resilience.

**IMPORTANCE:**

Lung and gut microbial diversity is significantly reduced in COPD patients. Oral microbiota is the primary source of lung microbes, but poorly predicts COPD status. *Haemophilus parahaemolyticus* was identified as a novel sputum biomarker in COPD. The bacterial network in COPD lungs is fragmented, lacking the resilience seen in healthy individuals.

## INTRODUCTION

Chronic obstructive pulmonary disease (COPD) is a prevalent chronic lung disease characterized by persistent airway inflammation and irreversible airway remodeling. COPD caused 3.3 million deaths in 2019 and is the third leading cause of death worldwide, posing a major health and economic burden (1). While cigarette smoking is a significant risk factor, with 15%–20% of persistent smokers developing the disease, genetic susceptibility and environmental factors (such as bacteria and viruses) may serve as “trigger factors” that drive and maintain chronic inflammation (2).

Microorganisms play a vital role in COPD pathogenesis, contributing to airway inflammation, mucus hypersecretion, and disease heterogeneity (3). Recent studies have revealed that bacterial diversity is reduced in the sputum of COPD patients, accompanied by an increased abundance of *Moraxella*, *Streptococcus*, *Veillonella*, *Haemophilus*, *Pseudomonas,* and *Prevotella* (4–6). In the airway microbiome, *Haemophilus* dominance has been associated with increased inflammatory responses, exacerbation frequency, and disease severity in COPD patients (7). The activation of interleukin (IL)-6 trans-signaling (IL-6TS) pathway caused by chronic colonization of *Haemophilus* may be an important disease driver in COPD patients (8).

The human body harbors a diverse microbial community that interacts through complex chemical signaling to maintain homeostasis and respond to environmental perturbations (9). When microbiome homeostasis is disrupted at one anatomical site, it can influence microbial communities and disease processes at distant locations through interconnected axes. For example, gut dysbiosis can affect lung immunity via the gut-lung axis, while oral pathogens can translocate to the lungs via the oral-lung axis, contributing to respiratory diseases (10).

Due to significant overlap of taxa between the oral, nasal, and pulmonary microbiota, the pulmonary microbiota is thought to originate primarily from the upper respiratory tract (11). Genera (such as *Prevotella*, *Streptococcus*, *Veillonella*, and *Neisseria*) commonly present in the oral cavity also predominate in healthy lungs (12). The respiratory tract exhibits a relatively homogenous microbiome, and healthy lungs do not possess a distinct microbiome separate from the upper respiratory tract (13). Previous studies have demonstrated that COPD patients harbor significantly higher proportions of certain oral bacterial genera, including *Desulfobulbus*, *Abiotrophia*, and *Selenomonas*, compared to the healthy group (14). Additionally, microbial community composition of the oral cavity and stomach shows the greatest similarity in COPD patients among various disease states, suggesting that oral microbiome dysbiosis may contribute to alterations in gastric microbiota through enhanced bacterial translocation or compromised mucosal barriers (15).

The gut microbiota of COPD patients had increased abundance of *Parasanguinis B* and *Streptococcus salivarius* (commonly found in the oral cavity), potentially reflecting oral-to-fecal microbial translocation (16). Despite these observations, the mechanistic basis and causal relationships underlying the oral-lung-gut axis in COPD remain poorly understood.

This study aims to characterize the composition, diversity, and biomarker taxa of oral, nasal, pulmonary (sputum), and gut microbiomes in patients with stable COPD compared to the healthy group, using full-length 16S rRNA gene sequencing. To identify microbial biomarkers associated with COPD across oral, nasal, sputum, and fecal samples and assess their correlations with clinical parameters (e.g., smoking, levels of IL-6, and C-reactive protein [CRP]) to elucidate their potential role in disease pathogenesis. This study aimed to investigate microbial interactions, network stability, and translocation patterns within the oral-lung-gut axis in COPD patients, focusing on the contribution of oral and nasal microbiota to lung and gut microbial communities.

## MATERIALS AND METHODS

### Subject registration

The Ethics Committee at Lanzhou University’s First Hospital approved the project (LDYYLL2023-421); the study adhered to the principles outlined in the Declaration of Helsinki, and all participants provided informed written consent. COPD patients were diagnosed by an experienced chest physician using spirometry and clinical examination. Diagnostic criteria included a forced expiratory volume in 1 second (FEV1)/forced vital capacity (FVC)  <70% and FEV1 percent predicted  <80%. COPD severity is classified according to the Global Initiative for Chronic Obstructive Lung Disease (GOLD) criteria based on post-bronchodilator FEV₁ percent predicted: GOLD 1 (mild, FEV₁ ≥80%), GOLD 2 (moderate, 50% ≤ FEV₁ <80%), GOLD 3 (severe, 30% ≤ FEV₁ <50%), and GOLD 4 (very severe, FEV₁ <30%) (17).

This study included 33 patients with COPD who were hospitalized in the department of respiratory medicine between December 2023 and December 2024, along with 29 healthy individuals recruited from the physical examination center. Clinical trial number: not applicable.

Inclusion criteria for the COPD group were as follows: (i) meeting the diagnostic criteria for stable COPD, patient’s clinical state remains stable for at least 30 days, with mild symptoms, and does not reach an acute exacerbation state requiring additional treatment intervention(18); (ii) no history of antibiotic use and systemic corticosteroid use within 1 month before admission; (iii) age over 40 years; and (iv) no other systemic diseases seriously affecting the physical condition.

Exclusion criteria included the following: (i) patients with other respiratory diseases, such as lung tumors, pulmonary tuberculosis, pulmonary fibrosis, etc.; (ii) those who could not cooperate with specimen collection; (iii) patients who had a history of infection within 1 month before admission and were treated with antibiotics.

### Sample collection

A clinical questionnaire was given to every participant, demographic information was noted, and samples were taken from the oral, nasal cavity, spontaneous or induced sputum, stool, and blood. Oral cavity: participants were instructed not to eat, drink, smoke, or chew gum for half an hour and wash their debris with drinking water. Use swabs to wipe all the corners of the mouth, including saliva, cheek mucosa, tongue, teeth, and oropharynx. Nasal cavity: A sample was taken from both nostrils (the anterior nares and posterior nasopharynx) through a cotton swab. Sputum samples were obtained using two methods: spontaneous coughing and induced sputum collection. Participants capable of spontaneous coughing first rinsed their mouths with sterile water and then coughed deeply, placing the sputum in a sterile container. The remaining participants underwent sputum induction via ultrasonic nebulization of 3% hypertonic saline for 20 min (19). To reduce visible saliva contamination, samples appearing to have high saliva content or viscosity were discarded. Within the COPD cohort, 17 individuals (51.5%) produced sputum spontaneously, while 16 (48.5%) had induced sputum; conversely, in the healthy group, eight participants (27.6%) coughed spontaneously, and 21 (72.4%) underwent sputum induction. A χ^2^ test revealed no significant variance in the proportion of induced versus spontaneous samples between these two groups (χ² = 3.673, *P* = 0.055). Approximately 5 g of feces sample was collected using a feces collection container, with the lid tightened and the sealing membrane closed. All specimens were collected within 24 h, placed in sterile frozen tubes, and frozen at −80°C. A total of 162 high-quality samples with sufficient DNA for sequencing were collected, 90 from COPD patients (COPDO: COPD oral, *n* = 30; COPDN: COPD nasal, *n* = 21; COPDS: COPD sputum, *n* = 19; COPDF: COPD feces, *n* = 20) and 72 from healthy group (CO, control oral, *n* = 28; CN: control nasal, *n* = 20; CS: control sputum, *n* = 11; CF: control feces, *n* = 13).

Blood samples were collected from all participants for hematological and inflammatory marker analysis. The following hematological parameters were measured using an automated hematology analyzer system (Sysmex XN-20; Kobe, Japan): white blood cell count (WBC), red blood cell count (RBC), hemoglobin (HGB), lymphocyte percentage (LYM%), monocyte percentage (MONO%), neutrophil percentage (NEUT%), eosinophil count (EO), and basophil count (BA). CRP levels were also determined using the same automated system. Body mass index (BMI) was calculated as weight in kilograms divided by the square of height in meters (kg/m²). Serum IL-6 levels were measured using electrochemiluminescence immunoassay (ECLIA) on a Roche Cobas e801 automated analyzer (Roche Diagnostics, Germany). The assay uses a sandwich immunoassay with biotinylated and ruthenium-labeled anti-IL-6 monoclonal antibodies. Streptavidin-coated microparticles capture the antibody-antigen complexes, which are magnetically attracted to electrodes. Electrochemiluminescence signals are generated by voltage application and measured by a photomultiplier. The assay has a detection range of 1.5–5,000 pg/mL and an analytical sensitivity of 1.5 pg/mL.

CRP and IL-6 were selected as representative systemic inflammatory markers because they are routinely measured in clinical practice, consistently elevated in COPD patients (20), and have been extensively associated with disease severity, exacerbation risk, and complications in previous studies (21–23).

### Full-length 16S rRNA gene sequencing

DNA was extracted using the TGuide S96 Magnetic Universal DNA Kit. To monitor and control for potential contamination in low-biomass samples, negative controls consisting of nuclease-free water (RT121, heat-sterilized) were processed alongside each batch of samples on every extraction plate. The negative controls underwent identical processing as biological samples through all downstream steps, including DNA extraction, PCR amplification, library construction, and sequencing. Polymerase chain reaction amplification was performed using fusion primers targeting the V1–V9 region of the 16S rRNA gene. The fusion primers used were 27F (5′-AGRGTTTGATYNTGGCTCAG-3′) and 1492R (5′-TASGGHTACCTTGTTASGACTT-3′) with the barcode. The products were purified, quantified, and homogenized to form a SMRTbell Library, and the qualified libraries were sequenced using PacBio Sequel II (Beijing Biomarker Technologies Co., Ltd., Beijing, China).

### Sequence data analysis

The original subreads were corrected to obtain Circular Consensus Sequencing (CCS) by using SMRT Link software (Version 8.0) with min Passes ≥5 and min Predicted Accuracy ≥ 0.9. Lima software (Version 1.7.0) was used to identify the CCS sequences of different samples through barcode sequences, cut the barcode sequences using cutadapt (Version 2.7), and then remove chimeras using UCHIME (Version 8.1) to obtain high-quality CCS sequences.

The Metaxa2 (Version 2.2.3) was employed to identify and extract bacterial 16S rRNA sequences while filtering out non-target sequences and potential contaminants. Only sequences confirmed as bacterial origin were retained for downstream Operational Taxonomic Units (OTUs) clustering. Additionally, taxa represented by fewer than 10 reads across the entire data set were excluded to minimize the impact of spurious low-abundance sequences.

The high-quality CCS sequences were clustered into OTUs with 97% similarity by using USEARCH (Version 10.0). The taxonomic identity of each OTU was annotated against the SILVA 138 reference database using the RDP Classifier method at a confidence threshold of 0.7. The OTUs belonging to the archaeal and unclassified categories were removed, and only bacterial OTUs were kept.

After the quality filtering process, a total of 8,438,605 high-quality CCS sequences were obtained from all samples (*n* = 162). The number of reads per sample ranged from 28,402 to 65,311. To standardize sequencing efforts across samples, each sample was normalized to a uniform count of 28,000 sequences. Following normalization, alpha diversity and beta diversity were calculated using R software (Version 4.3.3) with the Vegan package.

### Bioinformatics and statistical analysis

Demographic data were compared between groups using SPSS 27.0 (IBM, USA). Comparisons were conducted using a variety of statistical methods (including two independent-sample *t*-tests, the χ^2^ test, and the Fisher exact probability test). The Wilcoxon rank-sum test was used to compare bacterial abundance, alpha diversity, and beta diversity between COPD patients and the healthy group, as well as among different body sites. Linear discriminant analysis effect size (LEfSe) analysis (24) was performed to identify differentially abundant taxa between groups, with the default threshold of linear discriminant analysis (LDA) score >3.0 and *P* < 0.05. Spearman correlation coefficients between microbial taxa and clinical parameters were computed (25). To control for false discoveries arising from multiple hypothesis testing, *P*-values derived from Wilcoxon rank-sum tests and Spearman correlation analyses were adjusted using the Benjamini-Hochberg false discovery rate (FDR) procedure, implemented via the p.adjust function in R (method=“fdr”). The *q* < 0.05 was considered statistically significant.

The receiver operating characteristic (ROC) curves were utilized to assess the discriminative performance of microbial communities in distinguishing between health and COPD classifications. The migration rates of microorganisms from oral to nasal, oral to fecal, and nasal to sputum were calculated using the Source Tracker 2 software (26). All the graphs were plotted using R software with the ggplot2 package. Co-occurrence networks were constructed using the sparse correlations for compositional data method, with nodes selected based on a significance threshold of *P* < 0.05 and an absolute correlation coefficient of R^2^ >0.6. Network module analysis was performed using the "cluster fast greedy" algorithm, and Zi-Pi analysis as well as network attributes were evaluated. Network visualization was carried out using Gephi software. To assess the robustness of microbial co-occurrence networks, we performed a node removal simulation. Nodes were sequentially removed in descending order of degree centrality, and natural connectivity (λ̄), a metric quantifying network resilience based on adjacency matrix eigenvalues, was recalculated after each removal step (27). A steeper decline in natural connectivity indicates lower network robustness. Calculations were performed using the igraph package (v1.3.5) in R.

## RESULTS

### Characteristics of subjects

The average age of the participants was 66 years, of which 66.1% were male. There were no significant differences in age, gender, family history, and other general demographic data between the two groups (Table 1). The absolute counts of eosinophils also did not show any statistically significant difference. Compared with the healthy group, COPD patients had significantly higher rates of smoking (*P* = 0.032) and drinking (*P* = 0.037), which was statistically significant. The levels of two inflammatory indicators, CRP and IL-6, in the patients with COPD were also significantly higher; hence, the influence of poor environmental conditions (smoking and drinking) and blood inflammation levels on the microbiome cannot be excluded.

**TABLE 1:** Demographic information, blood indices and different inflammatory factors of participants^*a*^

		COPD	Healthy controls	*P* value
		(*N* = 33)	(*N* = 29)	
Age (year)		67.73 ± 7.84	64.76 ± 9.65	0.187
Nation(%)	Han nationality	32 (97.00)	25 (86.21)	0.176
	Other nationalities	1 (3.03)	4 (13.79)	
Gender	Male	23 (69.7)	18 (62.07)	0.527
BMI (kg/m^2^)		22.84 ± 3.59	23.52 ± 3.55	0.456
Smoking (%)		18 (54.55)	8 (27.59)	0.032
Drinking (%)		11 (33.33)	3 (10.34)	0.037
Hypertension (%)		13 (39.39)	10 (34.48)	0.690
Diabetes (%)		7 (21.21)	2 (6.90)	0.155
Coronary heart disease (%)		2 (6.06)	1 (3.45)	1.000
Family history of COPD（%）		4 (12.12)	2 (6.90)	0.676
CRP (mg/L) (0–4)		24.70 ± 36.63	3.89 ± 5.66	0.026
RBC (10^12^/L) (4.3–5.8)		4.91 ± 0.64	4.54 ± 0.84	0.088
HGB (g/L) (135–170)		150.57 ± 21.50	141.70 ± 20.82	0.159
WBC (10^9^/L) (3.5–9.5)		6.80 ± 2.28	5.99 ± 2.49	0.251
LYM% (20–50)		19.34 ± 11.78	24.25 ± 11.34	0.154
MONO% (3.0–10.0)		6.41 ± 2.16	7.30 ± 2.90	0.230
NEUT% (40–75)		71.95 ± 13.57	66.20 ± 12.36	0.140
EO (10^9^/L) (0.02–0.52)		0.12 ± 0.12	0.09 ± 0.06	0.437
BA (10^9^/L) (0–0.06)		0.02 ± 0.02	0.02 ± 0.01	0.854
IL-6 (pg/mL) (0–7)		48.65 ± 62.67	4.10 ± 1.80	＜0.001
FEV1/FVC		47.53 ± 12.72		
FEV1%		57.72 ± 23.94		
GOLD stage(%)	1	4 (12.12)		
	2	10 (30.30)		
	3	12 (36.36)		
	4	7 (21.21)		

^*a*^ BMI: body mass index; CRP: C-reactive protein; WBC: White Blood Cell Count; RBC: Red Blood Count; HGB: hemoglobin; NEUT%: neutrophil percentage; LYM%: lymphocytes percentage; MONO%: monocytes percentage; EO: eosinophils; BA: basophil; FEV1: forced expiratory volume in 1s; FVC: forced vital capacity. The *P*-value is calculated by two independent-sample t-test, Chi-Square Test, Fisher exact probability test.

### Core stable microbiota composition and individual changes

**Fig 1:**
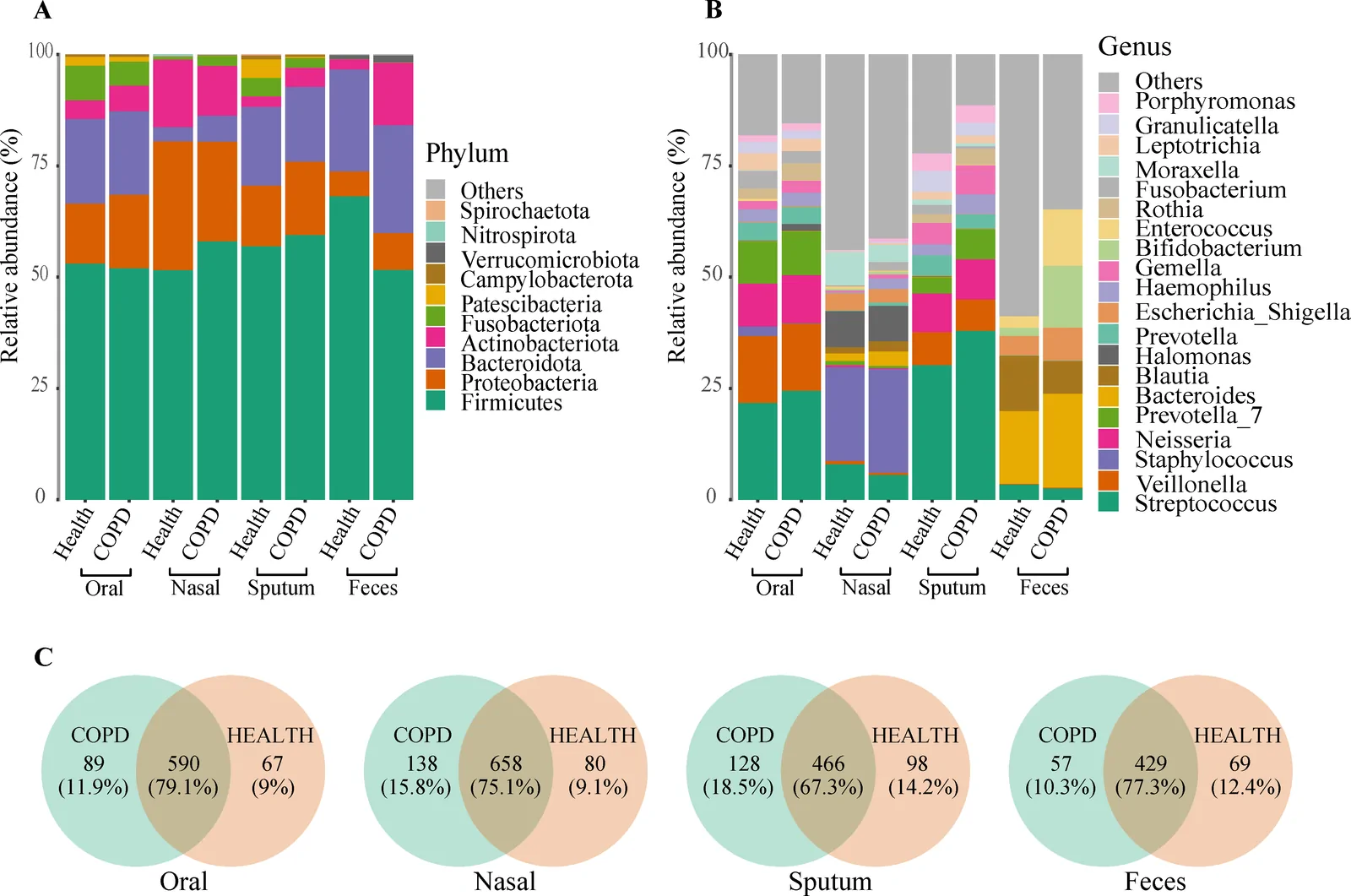
Community compositions of the microbiomes from different specimens in patients with COPD and the healthy group. (**A**) Stacked bar charts presenting the relative abundance of the top 10 bacterial phyla for each sample across four body sites. Samples are categorized by patients with COPD (COPDO, COPDN, COPDS, and COPDF) and the healthy group (CO, CN, CS, and CF). (**B**) Stacked bar charts depicting the relative abundance of the top 20 bacterial genera across the four body sites in both groups. (**C**) Venn diagrams demonstrating the number of shared and unique OTUs between patients with COPD and the healthy group at each body site (oral, nasal, sputum, and feces).

The dominant phyla across anatomical sites were *Firmicutes*, *Proteobacteria*, *Bacteroidota*, *Actinobacteriota*, *Fusobacteriota*, *Patescibacteria*, *Campylobacterota*, *Verrucomicrobiota*, *Nitrospirota*, and *Spirochaetota* at the phylum level (CO: 95.803%, COPDO: 99.998%; CN: 99.958%, COPDN: 99.922%; CS: 99.995%, COPDS: 99.988%; CF: 99.819%, and COPDF: 99.735%), (Fig. 1A). At the genus level, the top ten genera were *Streptococcus*, *Veillonella*, *Staphylococcus*, *Neisseria*, *Prevotella* 7, *Bacteroides*, *Blautia*, *Halomonas*, *Prevotella*, and *Escherichia_Shigella* (CO: 62.346%, COPDO: 65.982%; CN: 46.363%, COPDN: 47.224%; CS: 52.776%, COPDS: 60.899%; CF: 36.742%, and COPDF: 38.658%) (Fig. 1B). The oral cavity and sputum had similar microbial community structures, with *Streptococcus* being the most abundant. Accounting for 24.46% (oral cavity) in the COPDO group, 37.89% (sputum) in the COPDS, 21.69% in the CO group, and 30.19% in the CS group. Notably, *Haemophilus*, a genus predominantly associated with the oral cavity and respiratory tract, was also detected at low abundance in fecal samples and showed a significant difference between the two groups (*q* = 0.028), possibly reflecting ectopic gut colonization of oropharyngeal bacteria (Table S1). The Venn diagram indicated that COPD patients and the healthy group had overlapping OTUs of 590 in the oral cavity, 658 in the nasal cavity, 466 in sputum, and 429 in feces. Among the four groups, CF was the only group with a greater OTU count than COPDF (Fig. 1C).

### Alteration of the spectrum of microbial diversity

**Fig 2:**
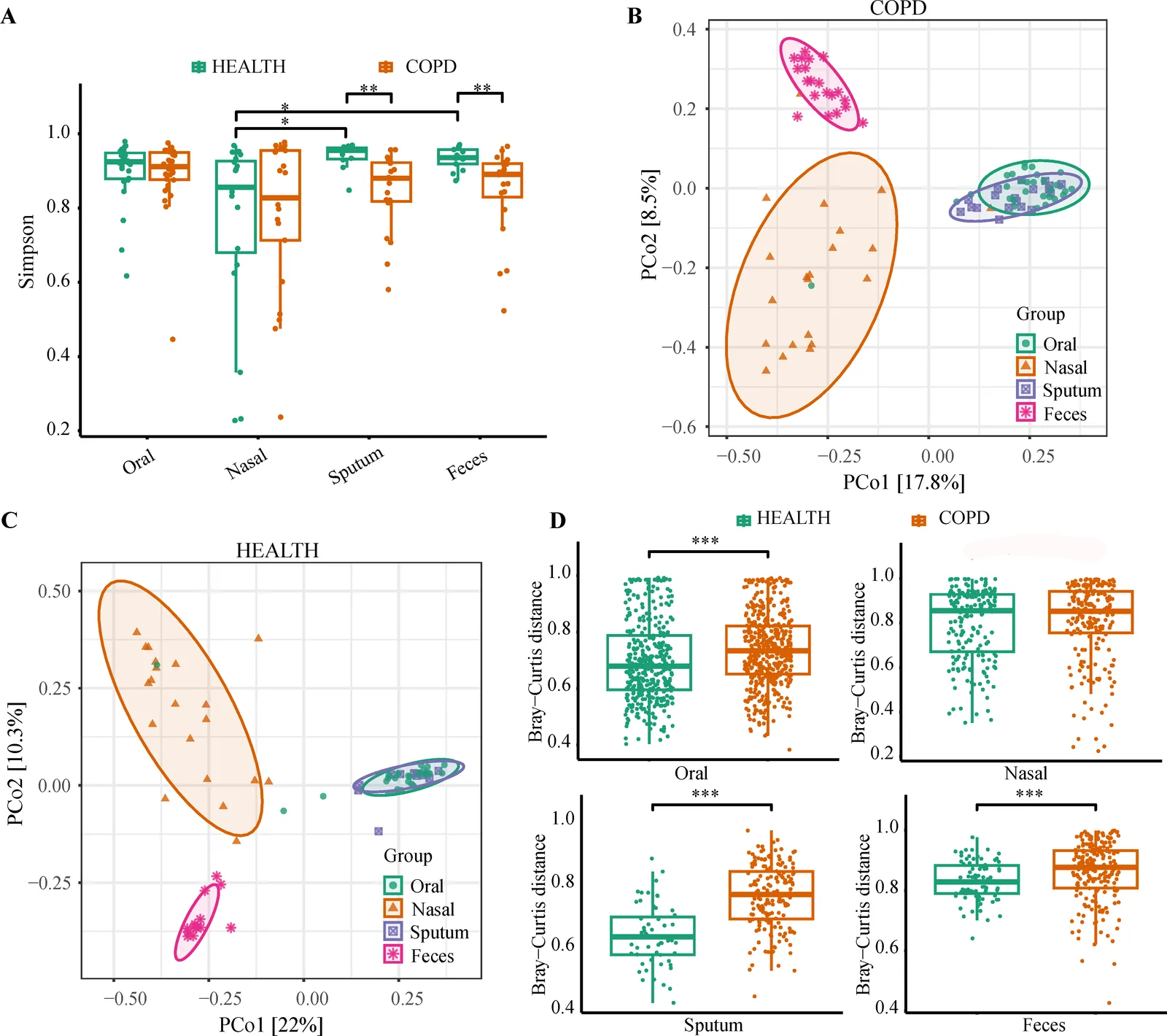
Diversity of microbiomes at the OTUs level. (**A**) Alpha diversity (Simpson index) was compared between patients with COPD and the healthy group at each body site: oral cavity (COPDO vs. CO), nasal cavity (COPDN vs. CN), sputum (COPDS vs. CS), and feces (COPDF vs. CF). Principal coordinate analysis (PCoA) based on Bray-Curtis dissimilarity at the OTUs level was conducted for (**B**) COPD patients and (**C**) the healthy group. (**D**) Box plots of pairwise Bray-Curtis distances were used to compare within-group microbial dissimilarity between COPD patients and the healthy group at each body site.

Nasal cavity microbial alpha diversity was the lowest in both COPD and the healthy group. In sputum and feces specimens, alpha diversity was significantly decreased in COPD patients (COPDS vs. CS, *q* = 0.004; COPDF vs. CF, *q* = 0.007) (Fig. 2A). However, oral samples did not show significant differences in alpha diversity. Higher inter-individual variability was observed in nasal samples compared with sputum and fecal samples, suggesting that nasal microbiota is susceptible to external environmental factors.

Principal coordinate analysis (PCoA) based on the Bray-Curtis distance metric was employed to investigate the variations in the relative abundance of OTUs across different bacterial groups. In both COPD patients and the healthy group, oral and sputum microbiota overlapped but were distinct from nasal and fecal microbiota (Fig. 2B and C). The Bray-Curtis distances of oral, sputum, and fecal samples in the COPD group were significantly higher than those in the healthy group (*q* < 0.001). Such findings suggest that the greater the dissimilarity in the microbial composition among samples within the COPD group, the more disordered the community structure and the stronger the heterogeneity (Fig. 2D). The COPD group exhibited a decrease in the Simpson diversity of its microbial community and an increase in heterogeneity in both sputum and feces.

### Identification of biomarkers in patients with COPD

**Fig 3:**
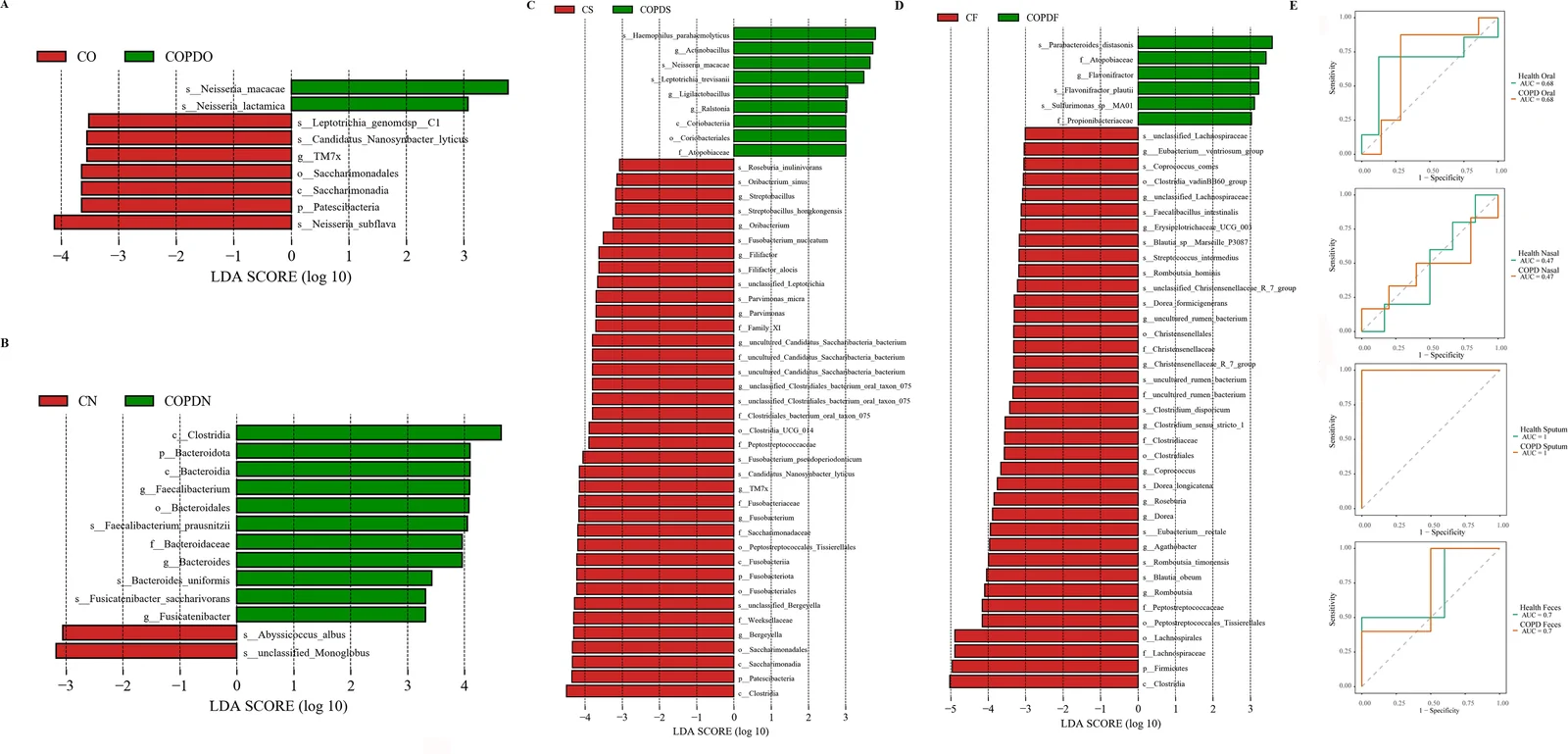
Identification of microbial biomarkers using the LEfSe method to distinguish COPD patients from healthy group across four body sites. (A–D) Linear discriminant analysis (LDA) effect size (LEfSe) analysis was conducted to identify differentially abundant bacterial taxa between COPD patients and healthy group (LDA score > 3.0, *P* < 0.05). (**A**) Oral cavity. (**B**) Nasal cavity. (**C**) Sputum. (**D**) Feces. (**E**) Mean test prediction accuracy was measured by the area under the ROC curve (AUC).

The linear discriminant analysis LDA was used to identify microbial biomarkers in four different anatomical sites in healthy control subjects and patients with COPD. At the species level, *Neisseria macacae* and *Neisseria lactamica* were recognized as biomarkers for the COPDO group (Fig. 3A). *Faecalibacterium*_*prausnitzii* is a biomarker for COPDN group (Fig. 3B). In the COPDS, *Haemophilus parahaemolyticus, Neisseria_macacae* and *Leptotrichia_trevisanii* served as biomarkers (Fig. 3C), and *Parabacteroides distasonis*, *Flavonifractor plautii,* and *Sulfurimonas_sp_MA01* are biomarkers for the COPDF group (Fig. 3D). The sputum bacteria can fully distinguish between patients with COPD and healthy group (area under the curve, AUC = 1.00), which is consistent with the respiratory disease characteristics of COPD. Oral and fecal bacteria exhibit moderate discriminatory ability (AUC ≈ 0.68–0.70). Nasal bacteria demonstrate nearly no discriminatory ability (AUC = 0.47) (Fig. 3E).

### Correlation with clinical information and inflammatory factors

**Fig 4:**
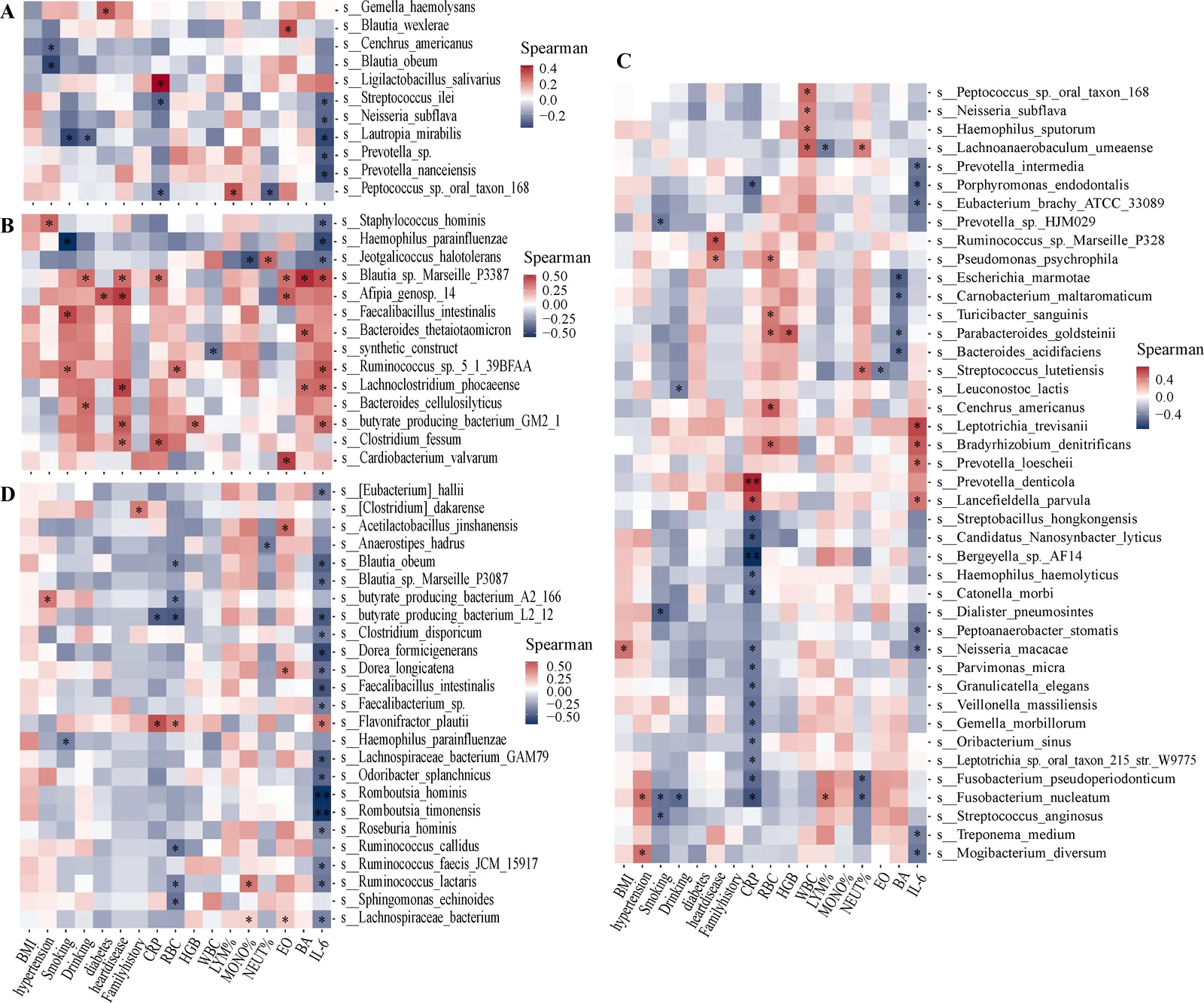
Spearman correlation heatmaps illustrate the relationship between microbial taxa and host clinical parameters. The colors of the heatmaps represent correlation coefficients (red: positive correlation, blue: negative correlation) for (**A**) the oral cavity, (**B**) the nasal cavity, (**C**) sputum, and (**D**) feces. Asterisks indicate the significance level after false discovery rate (FDR) correction (* *q* < 0.05, ** *q* < 0.01, *** *q* < 0.001). BMI: body mass index, CRP: C-reactive protein, RBC: red blood cell count, HGB: hemoglobin, WBC: white blood cell count, LYM%: lymphocyte percentage, MONO%: monocyte percentage, NEUT%: neutrophil percentage, EO: eosinophil count, BA: basophil count, IL–6: interleukin 6. All inflammatory markers were measured from peripheral blood.

To investigate disease-relevant associations, correlation analyses were conducted between clinical parameters and microbial taxa among the different anatomical sites. In the oral environment, CRP is positively linked to *Ligilactobacillus salivarius* (*q* = 0.042), and *Lautropia_mirabilis* is negatively related to smoking (*q* = 0.042), drinking (*q* = 0.038), and IL-6 (*q* = 0.042) (Fig. 4A). In nasal samples, there was a positive correlation of *Ruminococcus_sp._5_1_39BFAA* with smoking (*q* = 0.047), RBC (*q* = 0.048), and IL-6 (*q* = 0.046) (Fig. 4B). Sputum analysis showed that there was an inverse relationship between smoking, drinking, and *Fusobacterium nucleatum* (*q* = 0.044). The level of CRP is positively associated with *Prevotella_denticola* (*q* = 0.008) and is negatively associated with many bacteria, such as *Fusobacterium nucleatum* (*q* = 0.033), *Neisseria macacae (q* = 0.045*), Haemophilus haemolyticus (q* = 0.044*),* and *Veillonella massiliensis* (*q* = 0.022). The level of IL-6 exhibited a negative association with *Porphyromonas endodontalis* (*q* = 0.045) and *Prevotella intermedia* (*q* = 0.044) (Fig. 4C). The IL-6 in feces samples showed an adverse correlation with different microbiome representatives, including *Dorea_formicigenerans* (*q* = 0.018), *Romboutsia_hominis* (*q* = 0.003), butyrate-producing bacterium *(q = 0.024)*, *Faecalibacillus*_*intestinalis* (*q* = 0.024), and *Lachnospiraceae_bacterium* (*q* = 0.046) (Fig. 4D).

### 3.6 Microbial translocation and ecological network topology

**Fig 5:**
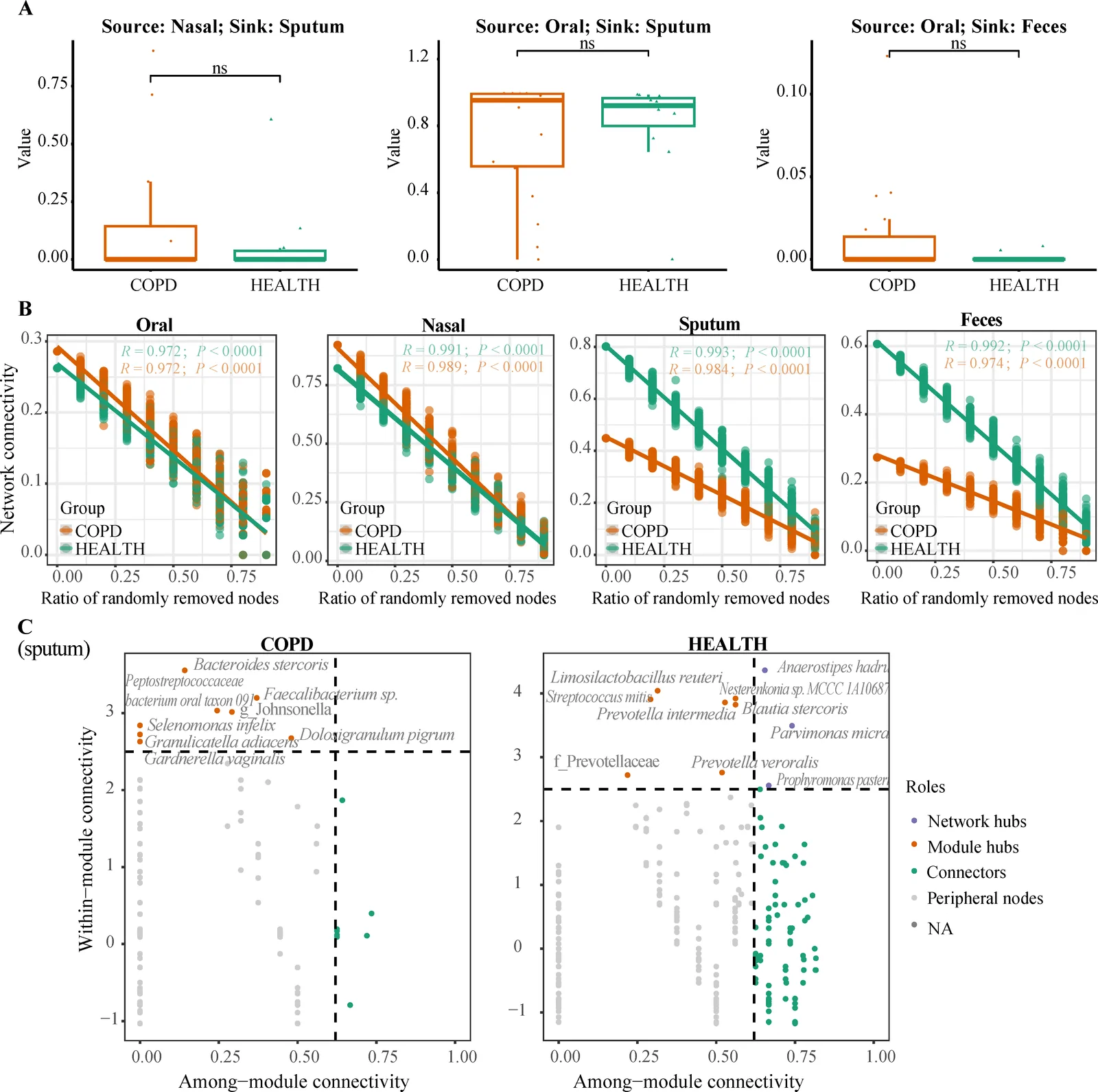
Interrelationship among microbiomes from different anatomical sites. (**A**) The proportions of oral/nasal bacteria migrating into the sputum and of oral bacteria migrating into the gut were estimated using Source Tracker software. (**B**) Network structural robustness was assessed by plotting network connectivity against the randomly removed nodes. (**C**) Zi-Pi plots were used to identify keystone taxa in the sputum microbial co-occurrence networks.

Source Tracker software was used to determine the contribution of oral/nasal microbes to the lungs in COPD patients. In COPD patients, 8.44% of lung microbes were from the nasal cavity, 73.87% from the oral cavity, and 2.43% of intestinal microbes come from the oral cavity. In the healthy group, 4.97% of the lung microorganisms found in the lungs originated from the nasal cavity, 80.97% were sourced from the oral cavity, and only 0.79% of the intestinal microbiome were derived from the oral cavity. The majority of microorganisms found in the lungs are derived from the oral region, and COPD patients showed more translocation of microorganisms from the nasal cavity to the lungs and from the oral cavity to the intestine than normal subjects, although there was no statistical difference between the two investigated groups of participants (Fig. 5A). In all four niches, the network connectivity decreased significantly with increasing node removal in both groups (all *P* < 0.0001), indicating progressive network fragmentation under perturbation. While oral and nasal microbiota showed similar robustness patterns between groups, marked differences were observed in sputum and fecal microbiota. Healthy group maintained higher overall connectivity throughout the removal process, whereas COPD networks exhibited consistently lower connectivity values. These results suggest diminished network complexity and reduced ecological stability in COPD-associated microbial communities (Fig. 5B). In sputum samples, the proportion of positive correlations in the network was greater than that of negative correlations, showing that microbial interaction in COPD lungs were mainly synergistic, and the synergy of the microbial community increases in response to lung disease (Fig. S2). To further characterize the structural organization of the sputum microbial networks, we classified nodes based on within-module connectivity (Zi) and among-module connectivity (Pi). In the COPD network, the majority of taxa were classified as peripheral nodes, with only a limited number of module hubs and connectors identified. Several taxa, including *Granulicatella adiacens*, *Selenomonas infelix*, *Dolosigranulum pigrum*, *Gardnerella vaginalis*, *Bacteroides stercoris*, *Peptostreptococcaceae bacterium oral taxon 091*, and *Faecalibacterium sp.*, were identified as module hubs, indicating locally important roles within specific modules. However, no prominent network hubs were observed, suggesting reduced global integration of the microbial network. In contrast, the healthy network exhibited a more complex topological structure. In addition to module hubs such as *Streptococcus mitis*, *Prevotella intermedia*, *Prevotella veroralis*, *Nesterenkonia* sp. *MCCC 1A10687*, *Limosilactobacillus reuteri*, and *Blautia stercoris*, multiple connector nodes were detected, bridging different modules. Notably, several taxa approached or exceeded the network hub threshold, indicating stronger inter-module connectivity and higher structural integration. Overall, the sputum microbiota in the healthy group exhibited a more interconnected and hierarchically organized network structure. (Fig. 5C).

## DISCUSSION

The current study characterized the microbiota across four anatomical sites in stable COPD patients and the healthy group using full-length 16S rRNA gene sequencing. Our results indicated reduced microbial diversity in sputum and feces, but not in oral or nasal samples of COPD patients. The lung microbiome was predominantly oral in origin; however, COPD altered its composition and network properties, and the network of COPD presented a more fragmented state, characterized by a smaller number of high-centrality taxa and diminished cross-module interactions.

We observed significant reductions in alpha diversity in sputum and fecal samples of COPD patients, consistent with previous reports (4, 6, 16, 28). In contrast, oral and nasal microbiota showed no significant differences in alpha diversity between groups (11, 29), although they exhibited higher inter-individual variability. This finding suggested that the oral and nasal microbiomes are more strongly shaped by host-specific and environmental factors, such as diet, hygiene, and smoking habits, than by COPD status (29, 30). This also explains that the composition and changes of the microbiomes in the lungs are dependent on changes in the oral and nasal cavities but do not come entirely from the upper respiratory tract. Indeed, the significantly higher smoking prevalence in our COPD cohort represents a potential confounding factor, and we did not make multivariate adjustments for smoking; hence, the observed differences in oral and nasal microbial communities should be interpreted with caution.

Source tracking analysis confirmed that the majority of sputum microbiota originated from the oral cavity in both COPD patients and the healthy group, with a smaller nasal contribution. The oral cavity as a reservoir for the respiratory, and the lung microbiome is primarily determined by the balance between oropharyngeal microbial migration, replication, and mucociliary clearance (31). Additionally, the spread of oral microbes to the lungs is heterogeneous, with more oral microbes entering the lungs being associated with decreased lung function and increased lung proinflammatory cytokines (32). The slightly higher nasal contribution in COPD patients (8.44%) may reflect impaired mucociliary function, potentially enhancing the binding of bacteria to the epithelium and promoting the colonization of pathogenic microorganisms (33, 34). Although the oral and sputum microbiomes are highly overlapped (34, 35), the accuracy of using oral microbiome changes to diagnose COPD was 68% in the ROC analysis. Therefore, the validity of using alterations in the upper airway as a biomarker for the evaluation of lower respiratory tract disease is still in doubt.

*Haemophilus influenzae* is a widely recognized cause of lower respiratory tract infections in adults, especially in patients with COPD (36). Interestingly, we identified *H. parahaemolyticus* as specific biomarkers for the COPD sputum group. *H. parahaemolyticus* is frequently overexpressed in lung diseases such as bronchiectasis (37) and nontuberculous mycobacterial lung disease (NTM-PD) (38) and is more likely to be present in patients who are stable on treatment and have no exacerbations (38). *H. parahaemolyticus* can compete with other bacteria to maintain the respiratory microbiome balance (39), which could also explain *H. parahaemolyticus* as a biomarker of stable COPD.

An interesting finding in this study was the detection of *Haemophilus* in fecal samples in COPD, despite it being primarily a respiratory tract commensal. This observation is consistent with the concept of ectopic colonization, whereby oral and respiratory bacteria translocate to the gastrointestinal tract; across all four niches, the network connectivity decreased significantly with increasing node removal in both groups (all *P* < 0.0001), indicating progressive network fragmentation under perturbation. Research indicates that *Klebsiella spp.* isolated from the salivary microbiota are strong inducers of T helper 1 (TH1) cells and elicited a severe gut inflammation (40). Oral bacterium, *H. parainfluenzae*, has been associated with Crohn’s disease (CD) severity and progression (41). The incidence of CD and ulcerative colitis (UC) is 55% and 30% higher in COPD patients than in the general population, respectively (42), indicating a continuum between inflammation in the upper and lower respiratory tract. We suppose that mucosal barrier function may be impaired in COPD patients, and *Haemophilus* in the oral cavity and respiratory tract may be more likely to transfer to the intestine, which leads to an increase in the relative abundance of *Haemophilus* in the gut.

We also evaluated the relationship between the microbiome and systemic inflammation, but their correlations were sparse. Several negative correlations were identified; IL-6 was inversely associated with *Porphyromonas_endodontalis* and *Prevotella_intermedia* in sputum, and with *butyrate-producing bacteria*, *Dorea_formicigenerans*, *Romboutsia_hominis*, *Faecalibacillus_intestinalis,* and *Lachnospiraceae_bacterium* in feces. These associations are consistent with the hypothesis that depletion of SCFAs-producing microbiota may contribute to systemic inflammation in COPD (43). It suggests that systemic inflammatory markers measured in peripheral blood may not be the primary drivers of the localized mucosal dysbiosis observed in stable COPD.

Co-occurrence network analysis of sputum microbiota revealed that COPD-associated networks were less robust than those of the healthy group. Furthermore, Zi-Pi analysis identified that the healthy network featured more prominent hub taxa with stronger inter-module connectivity. The shift of keystone roles toward low-abundance taxa may reflect a destabilized community in which dominant taxa have lost their organizational capacity in COPD (44). The overall structure of the microbial community and the occurrence of dysbiosis can be influenced by the presence of either low abundance of bacteria or by highly variable opportunistic pathogens (45, 46).

### Limitation

This study has some limitations. First, this is a cross-sectional study that involves a small number of subjects, making it difficult to establish a definitive causal link between COPD and microorganisms. Further studies with larger samples and cohort designs are needed. Second, the samples were collected from different parts of different patients, and the interference of confounding factors was inevitable. We utilized sputum as a representative sample for lung bacteria, and contamination by saliva and upper gastrointestinal contents cannot be ruled out. Spontaneous and induced sputum samples can vary in their cellular composition, the degree of upper respiratory contamination, and even their microbial characteristics. To mitigate this discrepancy, future studies should use standardized collection methods that are common to all participants.

### Conclusions

The microbial structures of the oral cavity, nasal cavity, lung, and intestines differ in COPD patients, and the diversity of sputum and feces microbiota decreases. Although the microbial structure in oral and sputum specimens is similar, changes in oral microbiota do not represent changes in lung microbiota. A fragmented ecological network in COPD may reflect a loss of community resilience, rendering the microbiota more susceptible to perturbation and pathogen invasion. The systemic inflammatory links to the oral-lung-gut axis appear limited and require further localized investigation.

## Data Availability

The datasets presented in this study can be found in online repositories. The names of the repository/repositories and accession number(s) can be found below: https://www.ncbi.nlm.nih.gov/bioproject/PRJNA1256799. The complete group classifications are publicly available from Zenodo (Liu, M. 2026) at https://doi.org/10.5281/zenodo.18767936.

## SUPPLEMENTAL MATERIAL

Supplemental material spectrum.00007-26-s0001.doc Fig. S1 and S2; Table S1.

ASM does not own the copyrights to Supplemental Material that may be linked to, or accessed through, an article. The authors have granted ASM a non-exclusive, world-wide license to publish the Supplemental Material files. Please contact the corresponding author directly for reuse.
