# Supplementary material for: Multi-site microbiomes’ response to chronic obstructive pulmonary disease
Source: Microbiol Spectr. 2026 May 18;14(7):e00007-26. doi: 10.1128/spectrum.00007-26 (PMC13340062; doi:10.1128/spectrum.00007-26)
Supplement: Supplemental material — Fig. S1 and S2; Table S1. [file spectrum.00007-26-s0001.doc]

**Table S1.** The relative abundance of each group in feces samples.

| Comparison | Genus | relative abundance (COPD group) | relative abundance (health group) | P.unadj | P. adj | Significance |
| --- | --- | --- | --- | --- | --- | --- |
| COPD VS Health (feces) | g__Blautia | 7.3480±1.9955 | 12.4283±1.4421 | 0.021 | 0.033 | * |
| COPD VS Health (feces) | g__Ruminococcus | 1.4766±0.6901 | 5.4033±1.8222 | 0.029 | 0.036 | * |
| COPD VS Health (feces) | g__[Ruminococcus]_torques_group | 0.4314±0.1369 | 3.7769±2.1333 | 0.020 | 0.033 | * |
| COPD VS Health (feces) | g__[Eubacterium]_hallii_group | 1.1966±0.5184 | 2.5272±0.6153 | 0.032 | 0.036 | * |
| COPD VS Health (feces) | g__Romboutsia | 0.3739±0.1809 | 3.5234±1.0564 | 0.000 | 0.003 | ** |
| COPD VS Health (feces) | g__Roseburia | 0.8943±0.5725 | 2.2706±0.6663 | 0.003 | 0.012 | * |
| COPD VS Health (feces) | g__Dorea | 0.4468±0.1955 | 2.3489±0.5380 | 0.001 | 0.005 | ** |
| COPD VS Health (feces) | g__Agathobacter | 0.2379±0.1228 | 2.6662±1.1627 | 0.003 | 0.012 | * |
| COPD VS Health (feces) | g__Anaerostipes | 0.5209±0.2634 | 1.6607±0.8581 | 0.004 | 0.012 | * |
| COPD VS Health (feces) | g__Clostridium_sensu_stricto_1 | 0.6691±0.3445 | 1.2536±0.5715 | 0.011 | 0.023 | * |
| COPD VS Health (feces) | g__Coprococcus | 0.1645±0.0809 | 1.2764±0.5592 | 0.004 | 0.012 | * |
| COPD VS Health (feces) | g__Erysipelotrichaceae_UCG_003 | 0.3450±0.1995 | 0.6310±0.1746 | 0.004 | 0.012 | * |
| COPD VS Health (feces) | g__Flavonifractor | 0.4223±0.1654 | 0.1915±0.1564 | 0.023 | 0.034 | * |
| COPD VS Health (feces) | g__[Ruminococcus]_gauvreauii_group | 0.1566±0.1137 | 0.3821±0.1550 | 0.028 | 0.036 | * |
| COPD VS Health (feces) | g__Monoglobus | 0.1023±0.0493 | 0.1948±0.0556 | 0.038 | 0.042 | * |
| COPD VS Health (feces) | g__Lachnospiraceae_NK4A136_group | 0.1190±0.0501 | 0.1039±0.0815 | 0.006 | 0.014 | * |
| COPD VS Health (feces) | g__[Eubacterium]_ventriosum_group | 0.0189±0.0093 | 0.1544±0.0425 | 0.000 | 0.003 | ** |
| COPD VS Health (feces) | g__Parasutterella | 0.0180±0.0117 | 0.0871±0.0458 | 0.004 | 0.012 | * |
| COPD VS Health (feces) | g__Odoribacter | 0.0129±0.0073 | 0.0896±0.0389 | 0.043 | 0.045 | * |
| COPD VS Health (feces) | g__Lachnospiraceae_ND3007_group | 0.0196±0.0151 | 0.0593±0.0378 | 0.031 | 0.036 | * |
| COPD VS Health (feces) | g__[Eubacterium]_ruminantium_group | 0.0000±0.0000 | 0.0885±0.0552 | 0.010 | 0.023 | * |
| COPD VS Health (feces) | g__CAG_56 | 0.0104±0.0091 | 0.0580±0.0318 | 0.016 | 0.029 | * |
| COPD VS Health (feces) | g__Haemophilus | 0.0021±0.0017 | 0.0066±0.0027 | 0.015 | 0.028 | * |
| COPD VS Health (feces) | g__Parvimonas | 0.0002±0.0002 | 0.0025±0.0016 | 0.047 | 0.047 | * |
| COPD VS Health (feces) | g__Sphingomonas | 0.0000±0.0000 | 0.0022±0.0013 | 0.029 | 0.036 | * |

Relative abundance values are presented as mean±SE. The comparison was conducted using the Wilcoxon Rank Sum test, and the p - value was validated using the FDR method.


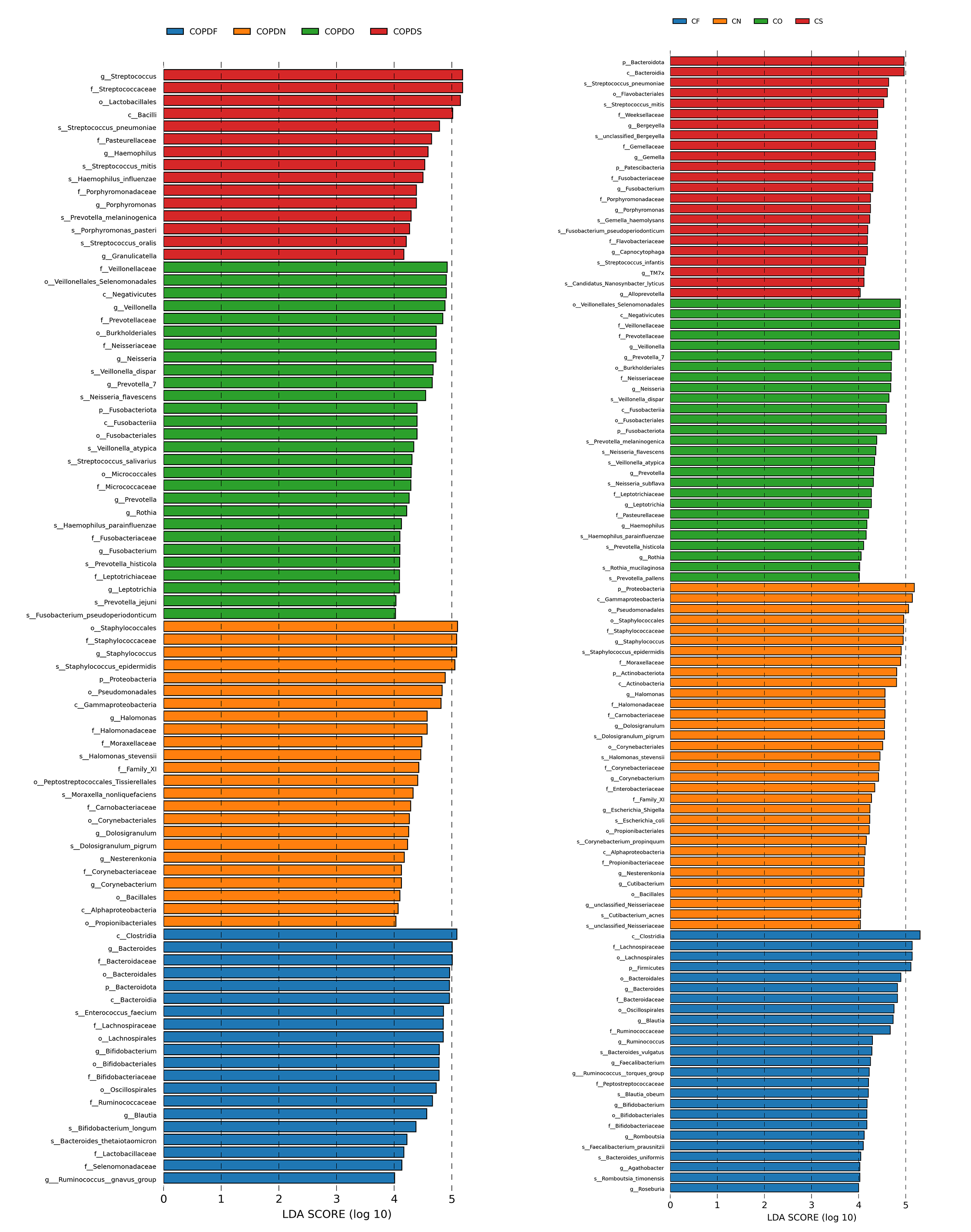


**Fig. S1.** Bacterial biomarkers in patients with COPD and healthy group across different anatomical sites. Taxa were selected based on a LDA score>4.0 and p<0.05. The LDA score bar plots present taxa that are enriched in COPDF/CF (blue), COPDN/CN (orange), COPDO/CO (green), and COPDS/CS (red) at multiple taxonomic levels, ranging from phylum to species.


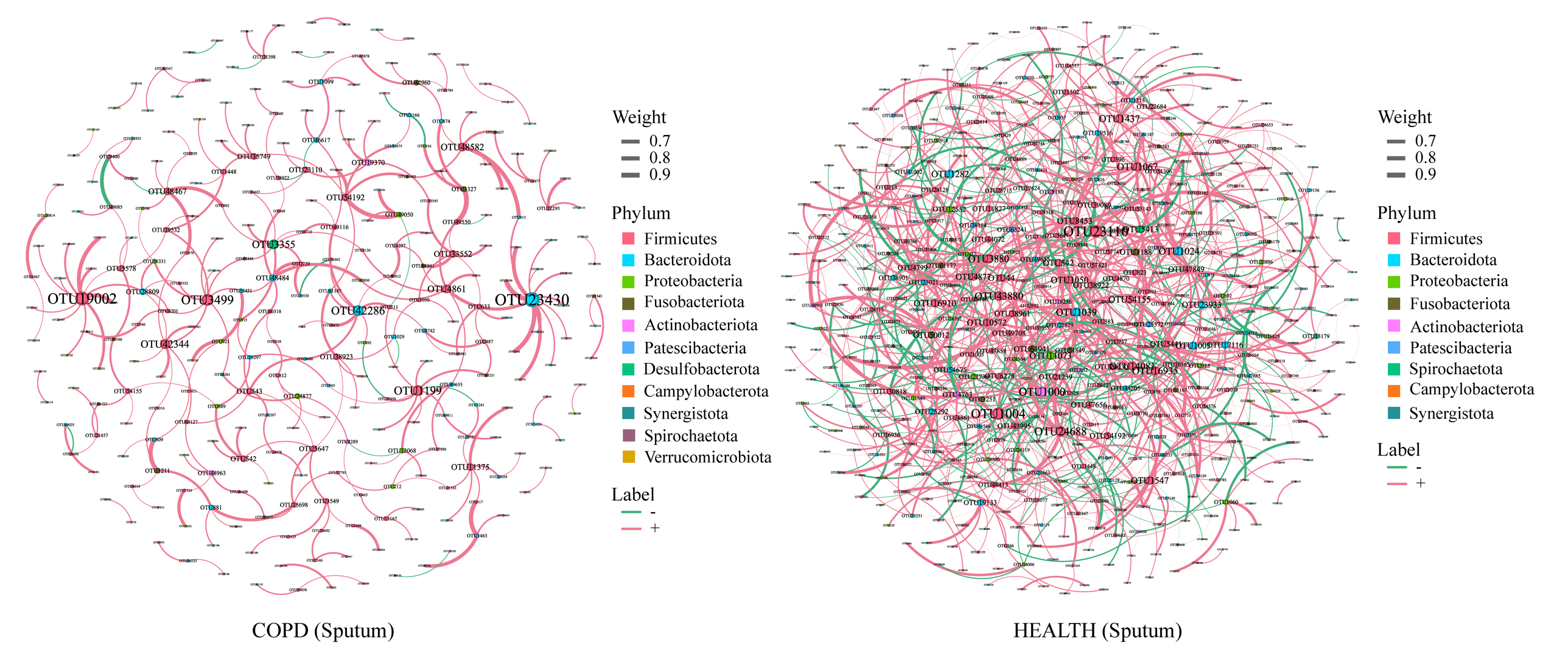


**Fig. S2.** The correlation network of sputum microbiota in COPD patients and healthy group. The co-occurrence network of sputum bacterial in patients with COPD and healthy group. Nodes represent bacterial OTUs. The color of each node represents the corresponding bacterial phylum, and the size of each node represents the degree of connectivity of the respective OTU. The color of the line represents positive (red) or negative (green) correlations, and the width of the line indicates the strength of the correlation.
